# Surgical Applications of Materials Engineered with Antimicrobial Properties

**DOI:** 10.3390/bioengineering9040138

**Published:** 2022-03-26

**Authors:** David P. Perrault, Ayushi Sharma, Jessica F. Kim, Geoffrey C. Gurtner, Derrick C. Wan

**Affiliations:** 1 Hagey Laboratory for Pediatric Regenerative Medicine, Stanford University School of Medicine, Stanford University, Stanford, CA 94305, USA; perrault@stanford.edu; 2 Division of Plastic and Reconstructive Medicine, Stanford University School of Medicine, Stanford University, Stanford, CA 94305, USA; ayushis@stanford.edu; 3 Department of Medicine, Loma Linda University School of Medicine, Loma Linda, CA 92350, USA; jessfkim@gmail.com; 4 Department of Surgery, University of Arizona School of Medicine, Tucson, AZ 85724, USA; gurtner@surgery.arizona.edu

**Keywords:** implant infection, surgical site infection, implant coating, antifouling, antibacterial coating, nanostructured surface, antibiotic implant, hydrogel

## Abstract

The infection of surgically placed implants is a problem that is both large in magnitude and that broadly affects nearly all surgical specialties. Implant-associated infections deleteriously affect patient quality-of-life and can lead to greater morbidity, mortality, and cost to the health care system. The impact of this problem has prompted extensive pre-clinical and clinical investigation into decreasing implant infection rates. More recently, antimicrobial approaches that modify or treat the implant directly have been of great interest. These approaches include antibacterial implant coatings (antifouling materials, antibiotics, metal ions, and antimicrobial peptides), antibacterial nanostructured implant surfaces, and antibiotic-releasing implants. This review provides a compendium of these approaches and the clinical applications and outcomes. In general, implant-specific modalities for reducing infections have been effective; however, most applications remain in the preclinical or early clinical stages.

## 1. Introduction

Infection is a large and impactful problem that affects nearly every surgical specialty. With implants, infection may be a particularly devastating complication. Implant infections can lead to hospitalization, the need for intravenous antibiotics, the surgical removal of the implant with multiple subsequent operations, and implant-specific complications. Cardiovascular implantable electronic device infections are well known to substantially increase morbidity and mortality [1]. With respect to the US health care system at large, infections are particularly costly. Surgical site infections may increase per-case expenditures by up to USD 22,667, thereby costing the healthcare system billions annually [2]. Surgical site infections and implant-associated infections remain a significant problem within surgery that impact patient morbidity, mortality, and add great cost to the health care system.

Given the burden of this problem, there have been multiple approaches to limit implant-associated infections. These include preoperative antibiotics [3], preoperative antiseptic preparation of the skin [4], antibiotic irrigation of the surgical site [5], and intra-operative warming [6]. Nonetheless, despite efforts to limit infections, this complication remains a problem. For implant-related infections, there are now several antimicrobial approaches that address the implant directly. Here, we review the approaches used to provide surgical materials with antimicrobial properties and provide examples of clinical use. The general methods employed to decrease surgical implant infections include specialized implant coatings [antifouling materials, antibiotics, metal ions, and antimicrobial peptides), antibacterial nanostructured implant surfaces, and antibiotic-releasing implants.

## 2. Implant Coatings

Treating the implant surface with antimicrobial material or modifying the implant surface is an obvious step to solving this problem. This approach focuses on the bacterial–implant interface and aims to either prevent biofilm formation, stop bacterial growth, or induce bacterial death.

### 2.1. Antifouling Coatings

**Figure 1:**
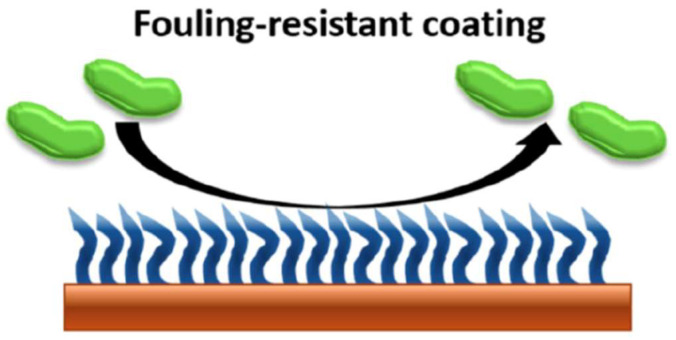
Schematic representing an antifouling coating. Reprinted with permission from Ref. [14] Copyright 2021 American Chemical Society.

Antifouling coatings work by limiting bacterial adhesion (Figure 1). The most common means of bacterial adhesion to implanted surfaces includes hydrophobic interactions and electrostatic charge [7,8]. As a result, frequently used coatings for antifouling are polymers with either hydrophilic or zwitterionic properties [9]. Hydrophilic coatings are well known to prevent the adhesion of hydrophobic bacteria, which have polysaccharide capsules [10]. Zwitterionic materials contain both positive and negative regions of charge but are overall electrically neutral. The strong dipoles of the zwitterion form a tight hydration layer and resist the deposition of bacteria on the surface [11]. Zwitterionic materials are also well known to resist bacterial adhesion [12]. The additional benefits of antifouling polymer coatings, in contrast to antibiotics, include the lack of promoting antibiotic drug resistance and the ability to function for prolonged periods of time [13]. 

Antifouling coatings have been applied clinically, most notably with urinary catheters, where hydrophilic catheters have been shown to reduce urinary tract infections among patients who require frequent intermittent catheterization [15]. It has also been shown that hydrophilic coatings effectively decrease the risk of infection in penile implants [16]. Other examples include dental implants, where antifouling hydrophilic coatings have been found to decrease bacterial adhesion in pre-clinical data [17]. Furthermore, improved osteointegration has also been described to be an added benefit with this particular application. However, no clinical data yet exist showing decreased infection rates with these coated dental implants [17]. Similar to hydrophilic antifouling coatings, zwitterionic materials have been studied extensively with promising antibacterial effects in multiple pre-clinical studies [12,18]. Clinically, however, zwitterionic coatings have yet to be shown efficacious in decreasing surgical implant infections. 

### 2.2. Antibacterial Coatings

Whereas antifouling coatings aim to prevent bacterial adherence, antibacterial coatings are designed instead to be either bacteriostatic or bactericidal. To accomplish this, a variety of antibacterial agents can be used to coat the implant. Notably, some of these coatings release the material into the implant microenvironment, while stable coatings keep the active compound bound to the implant. The less chemically stable coatings have the benefit of treating a larger area with the antimicrobial material; however, these coatings can cause local inflammation and have poorly controlled drug-release kinetics [19]. The antibacterial coatings reviewed here include antibiotic medications, antimicrobial ions/metals, and antimicrobial peptides (AMPs). 

#### 2.2.1. Antibiotic Medications

Implant coatings engineered with antibacterial medications have been heavily investigated for medical devices. In general, this typically involves loading a synthetic polymer, such as poly lactic-co-glycolic acid (PLGA), with an antibiotic drug [11]. By the nature of this design, these coatings are limited by variable pharmacodynamics, pharmacokinetics, polymer degradation proprieties, and the environmental properties in which they are implanted [11]. There are now a wide range of applications over a diverse set of surgical specialties, with a few pertinent examples discussed below. 

PLGA has been broadly applied with and without antibiotics in many different surgical procedures. One of the most common clinical applications of PLGA is its use as a resorbable implant in craniofacial surgery [20]. PLGA has excellent biocompatibility and resorbs over time, making the material an excellent candidate as a drug vehicle to coat surgical devices/implants [21]. Work to achieve consistent and sustained drug release is on-going, but promising [22]. A number of different antibiotics have been used to coat PLGA, including vancomycin, gentamicin, cefuroxime, and penicillin, and this construct has then been used to coat implants to effectively reduce microbial infections [23,24,25,26,27]. Preliminary studies have shown this strategy to be successful at preventing bacterial invasion and biofilm formation on implants [28]. Clinically, doxycycline-loaded PLGA has also been used as a treatment for chronic periodontitis in type-2 diabetics, resulting in decreased levels of inflammatory cytokines and microbial burden [29]. In a parallel, double-blinded, randomized, placebo-controlled clinical trial with 40 patients, Lecio et al. observed the use of doxycline-loaded PLGA nanospheres to decrease periodontal disease, with an associated reduction in interleukin-8 and interferon-gamma proinflammatory cytokine levels at 1, 3, and 6 months after treatment [29]. Locally applied antibiotic-coated PLGA may thus represent an encouraging adjunctive therapeutic for the management of local pathogens.

Antibiotic coatings have also been applied to stents for a variety of applications. Sinus stents, used to treat chronic infections, have been coated with ciprofloxacin, ciprofloxacin–ivacaftor, and ciprofloxacin–azithromycin and all have been shown to provide controlled antibiotic release, reduced bacterial load, and the limitation of biofilm formation [30]. Drug-eluting cardiac stents have revolutionized the field of interventional cardiology, most notably with medications such as paclitaxel and sirolimus, to prevent thrombosis and restenosis [31]. Antibiotics, such as rapamycin, have also been introduced into cardiac drug-eluting stents to limit restenosis, and this strategy has been shown to have similar efficacy to sirolimus-eluting stents [32]. Furthermore, endovascular stents for the treatment of mycotic aortic aneurysms are an obvious application given the infected surgical field. Endovascular stents coated with high-dose vancomycin can release antibiotics locally for up to 90 days and have been used successfully in selected patients [33,34].

Vascular graft infections are a rare but devastating complication. As such, some vascular prosthetics have been coated with slow-release antibiotics. This has been achieved by treatment with collagen, albumin, and gelatin to cross-link the antibiotics. In these coated devices, much of the antibiotic is released in the first 48 h, with elution continuing up to 7 days after placement, thereby providing antimicrobial protection in the immediate as well as acute recovery phase postoperatively [35,36,37].

Triclosan-eluting ureteral stents are another application of antibiotic-coated stents specifically aimed at reducing infection and other device-related complications. Triclosan, a polychloro phenoxy phenol with both phenol and ether functional groups, acts as a biocide at high concentrations and a bacteriostatic agent at lower commercial concentrations through the inhibition of bacterial fatty acid synthesis. Interestingly, in a clinical trial studying triclosan-eluting ureteral stents, no significant impact on infection was appreciated; however, the antibiotic stent reduced patient symptoms, including abdominal pain and urethral pain upon urination [38,39]. 

Surgical sutures have also been adapted and coated with antibiotics to limit bacterial colonization and to prevent infection [40]. Of note, while pre-clinical data have demonstrated minimal cytotoxicity, [41] antibiotic-coated sutures have not been shown to have a significant benefit in reducing postoperative infection. Scanning electron microscopy of *Staphylococcus aureus* and *Enterococcus faecalis* growing on surgical suture was noted by Henry-Stanley et al. to resemble bacterial biofilm [42], and triclosan coating has been found to be ineffective at inhibiting their growth [41]. A recent randomized controlled clinical trial looking at methods of reducing surgical site infections in low-income and middle-income countries found that triclosan-coated sutures were not beneficial in clean-contaminated or contaminated/dirty surgical wounds [43]. Importantly, with no significant difference in risk of infections between triclosan-coated versus non-coated sutures (relative risk 0.90), and given the higher cost of triclosan incorporation, the routine use of antibiotic-coated sutures has not been recommended.

Aside from these prefabricated, manufactured constructs integrating antibiotics, the coating of orthopedic implants with antibiotic cement is commonly performed by many orthopedic surgeons intraoperatively. Most frequently, vancomycin, gentamycin, and/or tobramycin is mixed into cements (e.g., polymethylmethacrylate (PMMA)) as it is formed. As the cement dries, naturally occurring imperfections containing the antibiotic release the compound into the local environment. However, controversy exists surrounding the pharmacokinetics of antibiotic delivery. While some studies have found the inadequate delivery of antibiotics from the cement and limited antimicrobial effect clinically [44], other reports have shown a good effect. Of note, gentamycin and ethylene glycol have been effectively bound to implants and have been shown to have good antibacterial activity against both *Staphylococcus aureus* and *Escherichia coli* [45]. Antibiotic cement-coated intramedullary implants have been demonstrated to decrease the need for additional procedures and allow for faster rehabilitation in patients treated for septic long bone nonunion [46]. Additionally, there are also pre-clinical data suggesting the improved osteointegration of antibiotic-coated implants, which is an obvious benefit for this application [47]. Notably, however, significant heterogeneity still exists in the literature regarding clinical outcomes, suggesting that more work needs to be done before the best method of coating orthopedic implants is known [48].

Finally, other alternatives to antibiotic cement have been explored for orthopedic implants. Specifically, ceramic coatings that contain hydroxyapatite or calcium phosphate have also been used to deliver antibiotics. This has the dual effect of antibiotic delivery as well as a rigid scaffold [27,48,49]. However, ceramic coatings comprising hydroxyapatite or calcium phosphate with integrated antibiotics have yet to be used clinically. 

#### 2.2.2. Metal Ions

Silver, zinc, and copper are all metal ions with known antibacterial properties [49,50,51]. In particular, silver possesses broad antimicrobial activity and is used in a wide range of medical devices. Silver nanoparticles have been introduced to the surface of titanium implants and have been effective in inducing bacterial cell death [21,49]. Silver coating has also been applied in vascular grafts, with several commercial products presently available [52]. Additionally, promising data have shown that silver-coated megaprostheses for femur reconstruction can decrease infection rates by almost half [53]. Similar to silver, zinc has been found experimentally to have a strong antimicrobial effect when used as an implant coating [51,54]. However, zinc coating for the explicit purpose of antimicrobial effect has yet to be used clinically. Lastly, copper has been shown to kill *Escherichia coli* on contact, and although the mechanism of action is not well elucidated, it remains a promising implant coating for broad-spectrum antimicrobial coverage. Nonetheless, like zinc, copper coatings can be readily found commercially in various non-medical-grade devices, but clinical application with implants has yet to be described.

#### 2.2.3. Antimicrobial Peptides

**Figure 2:**
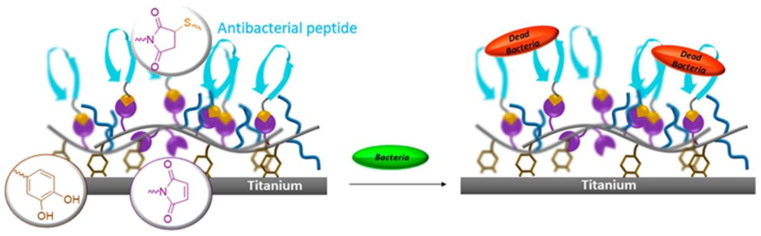
Schematic of antimicrobial peptides causing bacterial cell death. Reprinted with permission from Ref. [62] Copyright 2019 American Chemical Society.

Antimicrobial peptides (AMPs) are a component of the innate immune system and possess broad-spectrum antimicrobial activity (Figure 2). Over 300 AMPs have been described, seven of which have FDA approval for use [55]. Polymyxins, frequently used AMPs, are cyclic polypeptides produced by *Paenibacillus polymyxia* which bind to the outer membrane of Gram-negative bacteria, thereby increasing cell permeability, leading to death [56]. Daptomycin, another clinically used AMP, is naturally produced by *Streptomyces roseosporus* and inhibits bacterial cell wall synthesis [57]. Both have been shown in clinical trials to resolve skin infections comparable to standard-of-care antibiotics such as nafcillin or vancomycin [58]. Other ongoing clinical trials evaluating AMPs are primarily focused on the use of these peptides to treat severe infections or to reduce inflammation [59]. Notably, AMPs have only been studied for topical, oral, and intravenous delivery. Despite the present absence of any clinical applications integrating AMPs into an implant coating, there are robust pre-clinical data suggesting they may have a role in preventing implant infections in the future. Chen et al. demonstrated the integration of azido-AMPs to the surface of titanium implants to exhibit stable antimicrobial activity, inhibiting 90.2% of *Staphylococcus aureus* and 88.1% of *Escherichia coli* after 2.5 h of incubation [60]. In vivo implantation of this same AMP-coated construct was found to still be capable of killing 78.8% of *S. aureus* after 7 days in rabbits [60]. Importantly, azido-AMP-coated titanium implants were noted to have negligible cytotoxicity on mouse bone marrow mesenchymal cells. However, the long-term cytotoxic effects on human cells by AMPs requires further study before these antimicrobial peptides may be more widely adopted [61]. 

## 3. Nanostructured Surfaces

Nanostructures are constructs that range between 1 nm and 100 nm and therefore exist on the molecular scale in at least one dimension. Alterations at the nanostructure level have been shown to impart the beneficial antibacterial properties of many materials. Interestingly, naturally existing nanostructures have served as inspiration for designing surfaces with antimicrobial function, with examples coming from the lotus leaf and cicada wing. 

The lotus leaf (*Nelumbo nucifera*) is superhydrophobic, possessing a water contact angle greater than 160° and a sliding angle less than 5° to prevent the collection of water droplets, dirt, and debris [63]. Scanning electron microscopy of the surface has revealed low-surface-energy epicuticular wax crystalloids 1–5 μm in height; air trapping between wax crystalloids and beneath the floating water droplets contribute the self-cleaning nature of the lotus leaf [64,65]. Similar nanostructural surface modifications have been engineered based on the lotus leaf for clothes, windows, paint, and low-friction surfaces. Additionally, these nanostructures have been shown to prevent bacterial adhesion in a variety of conditions [66]. Materials designed with superhydrophobicity similar to the lotus leaf have been demonstrated to prevent or reduce the attachment of bacterial strains such as *Staphylococcus aureus* and *Pseudomonas aeruginosa* [67,68]. However, bacterial repulsion based on hydrophobicity is complex and generally remains poorly understood, as certain Gram-positive microbes have been found to adhere well to this surface.

**Figure 3:**
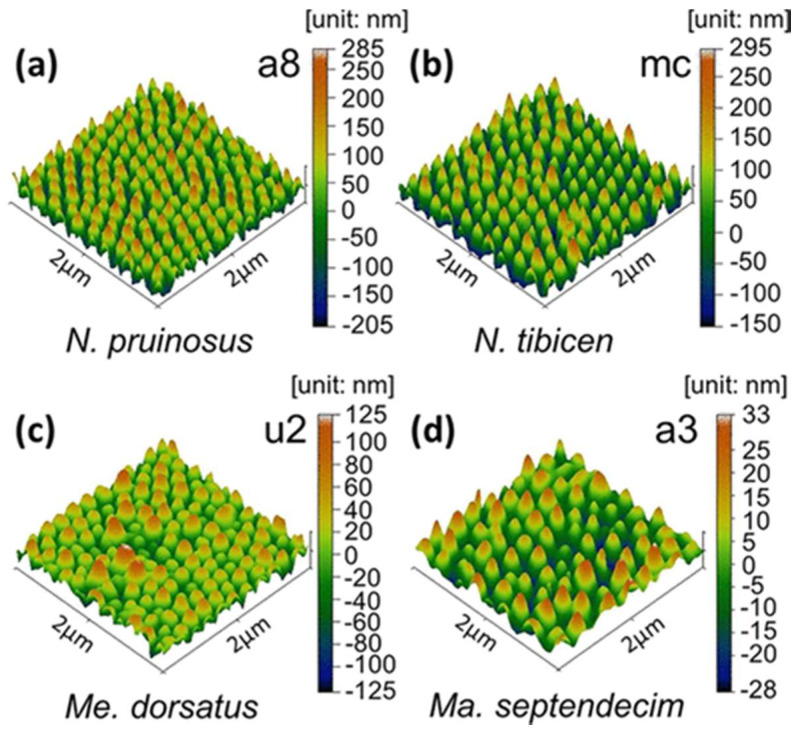
Representative scans of various species of cicada depicting the nanopillar structure, including (**a**) N. pruinosus, (**b**) N. tibicen, (**c**) Me. Dorsatus, (**d**) Ma. Septendecim. Reprinted with permission from Ref. [73] Copyright 2017 American Chemical Society.

Much like the lotus leaf, the composition and structure of the cicada wing also lends itself to a high surface hydrophobicity (Figure 3). Cicadas are a family of insects with membranous wings consisting of chitin, wax, and protein with nano-pillar structures 150 nm in height [69,70]. While this feature may limit the adherence of some bacteria, studies have also demonstrated a bactericidal capacity. Ivanova et al. found that contact with cicada wing surfaces kills *P. aeruginosa* within three minutes [71]. Modeling of the cicada nano-pillar structure has revealed cellular membrane rupture to contribute to Gram-negative bacterial death [71]. Nonetheless, despite very promising in vitro evidence in support of antibacterial nanostructures that mimic the lotus leaf and cicada wing, this technology has yet to be clinically translated [72].

There has also been effort to design dental implants with antimicrobial properties given that implant-related infection is one of the leading causes of implant failure [74]. One promising modification to standard titanium implants is treating the surface to become more rough on the order of its nanostructure. This can be achieved with sandblasting and acid etching [75,76,77,78]. Bierbuam et al. found that sandblasted/acid-etched surfaces had a reduction in bacterial adhesion of 61% compared with standard titanium surfaces [75]. A point of concern for modifying the dental implant surface in particular is to not compromise the osteointegration of the implant. Reassuringly, Marconi et al. showed that double acid etching a titanium surface improved a number of metrics thought to improve osteointegration [78]. These include the improved adhesion capacity of human peridontal ligament stem cells (hPDLSCs) onto the titanium surface, as well as the over-expression of fibronectin, laminin, N-cadherin, and RUNX2 in PDLSCs.

## 4. Antibiotic-Releasing Implants

The treatment of infections, particularly in avascular tissues, can be difficult due to limited delivery of systemic antibiotics [79]. Controlled release implants may have the potential to generate a higher local concentration of antibiotics with reduced systemic toxicity [28,80]. While antibiotic coatings to limit metallic implant infection have been previously discussed, toward a goal of infection treatment many antibiotic-releasing materials have been developed, including antimicrobial beads, biofilms, cements, micro-reservoirs, and other antibiotic carriers such as hydrogels. 

Antibiotic cements, reviewed above in the context of coating orthopedic implants, as well as other bone substitutes, can also be used independent of metallic implants as a drug delivery system. Bone cements, such as polymethylmethacrylate impregnated with antibiotics, have been widely used in both orthopedic and trauma surgery. In addition to coatings, freely mobile spacer beads that contain an antibiotic depot can be placed around the implant. Bone cements and other substitutes impregnated with antibiotics and antibiotic-containing spacer beads have also been employed in the treatment of osteomyelitis seen with diabetic foot infections. Whisstock et al. conducted a retrospective study in which a resorbable gentamicin-loaded bone graft substitute comprising calcium sulphate and hydroxyapatite was used in the treatment of osteomyelitis that developed secondary to diabetic foot infections. In that study, the gentamicin-eluting implants were found to adequately manage dead space following bone debridement, and an overall cure rate of 81.3% was observed among 35 patients at one-year follow-up [81].

Within cardiovascular surgery, cardiac mesh envelopes used to wrap implantable electronic devices are an exciting development. Similar to the use of antibiotic beads as a drug delivery system, these devices also add an independent drug delivery depot to the implant space. TYRX™ (Medtronic Inc., Eatontown, NJ, USA) is a synthetic mesh envelope composed of large-pore tyrosine-based polymer filaments designed to hydrolytically degrade over nine weeks and impregnated with minocycline and rifampin antibiotics, which are released during this process. Use of TYRX^TM^ in the WRAP-IT trial demonstrated a 40% reduction in major infections and 61% reduction in pocket infections related to the placement of the cardiac implantable electronic device [82,83,84]. Based on these findings, the European Heart Rhythm Association has recommended use of TYRX^TM^ in patients undergoing initial defibrillator implantation, those undergoing pocket or lead revision, and in those with other high-risk factors such as the need for dialysis or immunosuppressive medications [85]. Other commercially available biologic materials can also be hydrated and impregnated with antibiotic solutions; this has been studied specifically with the CanGaroo-G™ device (Aziyo Biologics, Silver Spring, MD, USA), which consists of decellularized extracellular matrix. Originally designed as an envelope to minimize the migration of a cardiac-implantable electronic device [86], this has since been shown to also effectively deliver gentamicin [87]. Preclinical studies with the CanGarro-G^TM^ device have suggested improved tissue integration, better vascular ingrowth, a lower inflammatory response, and the more rapid clearance of bacteria when compared with the TYRX^TM^ envelope [87].

The infection of implants that are in continuity with the central nervous system can lead to devastating complications, including encephalitis and meningitis. Ventriculoperitoneal (VP) shunt insertion for the treatment of hydrocephalus is one of the most common neurosurgical procedures [88]. Ventriculoperitoneal shunt insertion involves placing a silicone catheter that drains cerebrospinal fluid from the ventricles into the peritoneal cavity [89]. Infection of the silicone shunt is one of the most common reasons for revision surgery and shunt failure. Fernandez-Mendez et al. performed a retrospective multicenter registry study evaluating 41,306 procedures in 26,545 patients with VP shunts, and found that the infection rate of VP shunts was 16.5% [90]. Moreover, in a retrospective review of 1173 pediatric patients with VP shunts, the mortality related to shunt infection was 10.1%, and shunt infection was an independent negative predictor of school performance [91]. VP shunt infection is clearly a pertinent and impactful problem. Antibiotic-impregnated shunts have been used clinically to decrease the risk of infection. The BASICS trial, a multicenter single-blinded randomized study, evaluated the efficacy of antibiotic shunts (impregnated with 0.15% clindamycin and 0.054% rifampicin) in decreasing infection in 3505 patients [86]. In those receiving their first permanent VP shunt, infection rate was 2% with antibiotic shunts, 6% with standard shunts, and 6% with silver shunts. The lower infection rate with antibiotic shunts compared with standard and silver shunts was statistically significant [86]. Of note, silver-impregnated silicone shunts were also evaluated in the BASICS trial, but were not found to be effective in reducing infection rates. 

Silk-based biomaterials have similarly been explored for their use as antibiotic delivery devices. Silk microspheres have been loaded with antibiotics such as ampicillin and have been found to be effective in treating murine infected wounds. In a study by Pritchard and colleagues, compared with the direct injection of ampicillin, *S. aureus*-infected murine wounds managed with ampicillin-releasing silk microspheres were noted to have a significantly greater reduction in bacterial colony-forming unit counts [92]. Corresponding in vitro studies have shown sustained drug release overtime, with the ability to control drug delivery time from days to years via the manipulation of the crystallinity of the silk’s biochemical structure [92]. These findings highlight the potential for antibiotic-impregnated silk biomaterials, with their tunable delivery profile, to be further explored clinically as a focal delivery device to treat local infections.

Finally, crosslinked polymeric hydrogels for antibiotic delivery have been developed using a wide variety of synthetic and biologically occurring substances. Natural polymeric materials frequently employed in hydrogel preparation include collagen, tendon, chitin/chitosan, alginate, and hyaluronic acid (HA). Hydrogels are highly absorbent, maintain their structure, are biocompatible, and can be loaded with many medications which can be steadily released [93,94,95,96]. Furthermore, several hydrogels have been FDA approved and have broad applicability in the biomedical arena [30]. These qualities make hydrogels a great candidate for drug delivery. 

Nanoparticle-loaded biological hydrogels are three-dimensional structures with the capacity to hold a set amount of liquid and particle. Cross-linkage of the hydrogel materials prevents degradation and maintains stability in a dynamic and aqueous environment. Hydrogel antibiotic delivery is performed via two methods: bulk-loaded hydrogels and antibiotic-carrying microspheres suspended within the hydrogel. Bulk-loaded hydrogels provide a rapid release of antibiotic while microsphere hydrogels allow for more controlled and sustained release [92,97]. 

Antibiotic-loaded hydrogels are an exciting development with a broad scope of application [98,99]. Li et al. recently described the development of an “intelligent” hydrogel constructed of chitosan and hyaluronic acid loaded with vancomycin [100]. Hyaluronidase secreted by *S. aureus* triggers the release of vancomycin, and this has been shown to prevent bone infections in a rabbit model [100]. Smart hydrogels have also been designed with tunable on-demand drug release for local antibiotic delivery. Hu and colleagues described the fabrication of a dextran, cellulose, alginate, and chondroitin polymer hydrogel crosslinked using aminoglycosides [101]. Importantly, the hydrogel modulus and antibiotic release profile could be precisely tailored by varying the pH and the antibiotic dose during gel formation. Subcutaneous injection of this amikacin-loaded hydrogel in mice was found to effectively treat *S. aureus* infections. Furthermore, sustained amikacin release was observed, with high local antibacterial activities still detected two weeks following hydrogel injection [101]. The evolution of hydrogels has now led to the creation of dual-responsive, pH- and reactive-oxygen-species-sensing constructs designed to achieve specific drug release at sites of inflammation. These hydrogels, created by grafting phenylboronic acid to the side chain of alginate polymers, have been loaded with both amikacin and naproxen micelles [102,103]. In vitro and in vivo experiments have shown this to be effective at inhibiting the growth of both *S. aureus* and *P. aeruginosa* [102]. Of note, the anti-inflammatory drug naproxen was also released within 24 h under conditions with pH < 5.0 and 10 mM H_2_O_2_, reducing the levels of pro-inflammatory cytokines such as tumor necrosis factor-alpha [102]. Collectively, these studies highlight the potential for smart hydrogels to respond to local environmental cues and adeptly treat infections.

**Figure 4:**
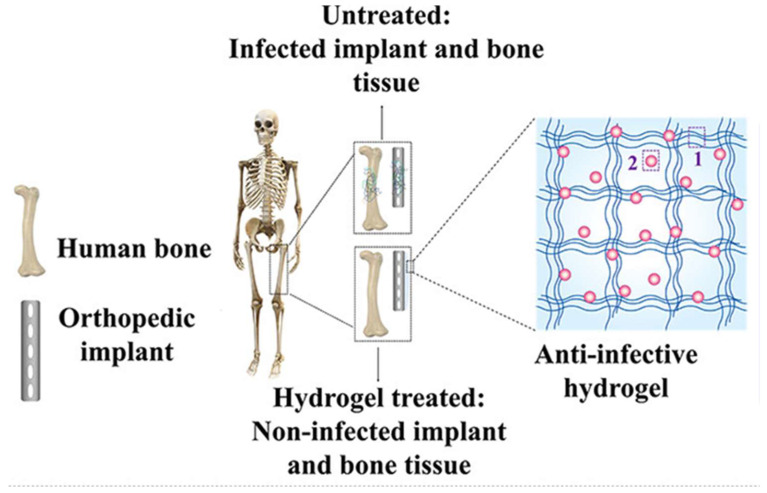
Schematic of hydrogel-treated orthopedic implants preventing infection. 1 represents the hydrogel polymer and 2 representes the antibiotic material within the hydrogel. Reprinted with permission from Ref. [108] Copyright 2021 American Chemical Society.

Over the last decade, the clinical use of antibacterial hydrogels has been increasingly reported (Figure 4). In the setting of periprosthetic joint injections, Pellegrini and colleagues reported on the ability for antibacterial hydrogels to facilitate one-stage revision total hip arthroplasty without the additional use of cement [104]. Among ten patients presenting with hip implant infections, DAC^®^ hydrogel (Novagenit Srl, Mezzolombardo, Italy) loaded with 5% gentamicin and 5% vancomycin was spread over the new implant surface prior to placement, and mean follow-up at 3.1 years revealed no clinical or radiographic signs of recurrent infection [104]. This compared favorably to one- or two-stage antibiotic-loaded cement approaches, which have been described to yield infection eradication rates up to 82% [105,106]. The same hydrogel has also been shown to effectively prevent early periprosthetic infections when used with joint megaprostheses. In a multi-center trial with 43 patients, no infections were identified at two years among patients receiving a joint megaprosthesis with antibiotic-loaded DAC^®^ hydrogel, compared with six cases of post-surgical infection in the control group [107].

A second commercially available antibiotic-loaded hydrogel has also been described for use with the ETN PROtect intramedullary tibial nailing system (DePuy Synthes, Zuchwil, Switzerland). In contrast to the DAC^®^ hydrogel, this second hydrogel consisting of absorbable poly (d, l-lactide) matrix with incorporated gentamicin sulphate is already directly applied to the implant during production [109,110]. Studies have shown 80% of antibiotic release within the first 48 h following implantation, followed by a controlled secondary timed release with breakdown of the polymer [111]. Clinical reports have demonstrated this hydrogel to be effective at preventing infections in high-risk patients with polytrauma, open fractures, or immunosuppression, as well as in the setting of infection related to revision surgeries [112].

Lastly, it is worth noting that a considerable amount of work has been carried out with respect to the wound dressings engineered with antimicrobial properties. Hydrogels, by themselves, are already in routine use for wound care, but the availability of dressings loaded with antibiotics or AMPs has the potential to revolutionize the management of many wounds. While a full review of this topic is far beyond the scope of this review, we note, however, that hydrogels in particular are especially promising as both a wound dressing and as a vehicle for antimicrobial or cellular therapies [113,114]. Using an animal model for burn wound infection, Zhu and colleagues found that the incorporation of colistin, a potent lipopeptide, into a chitosan-based hydrogel was effective at killing multidrug-resistant *P. aeruginosa* [115]. Likewise, amoxicillin has been incorporated into *N*-carboxyethyl chitosan and aniline conjugated hyaluronic acid hydrogels and this has been observed to kill both *S. aureus* and *Escherichia coli* in infected mouse wounds [116]. Antibiotic-loaded chitosan hydrogels have even been described to accelerate wound healing, with histologic analyses of hydrogel-treated mouse wounds demonstrating enhanced collagen deposition, blood vessel formation, and epithelial regeneration [117]. These studies, while preclinical, nonetheless underscore the promise for the use of antibiotic-loaded hydrogels to more effectively prevent or treat infected burns and to promote the regeneration of surgical and traumatic wounds.

## 5. Conclusions

Infection after surgery, especially with implants, can have a devastating impact on patient quality of life and remains a large burden on the US health care system. In light of this, a wide variety of methods have been developed by which to impart antimicrobial properties to surgical implants. The majority of these methods are still well within the pre-clinical phase. However, there are promising data to suggest that more effective modalities than are currently available will be developed for preventing surgical and implant-related infections. The strategies that have been applied clinically have had a mixture of outcomes, but in general appear to be effective in reducing the infectious complications of surgery. Ultimately, using these materials clinically at this stage will require a patient-specific approach and careful consideration.
